# The Effects of a Novel Fortified Dairy Product on Weight Loss, Metabolic Profiles, and Endocrine Hormones in Women With Polycystic Ovary Syndrome: A Double‐Blind Randomized Controlled Trial

**DOI:** 10.1002/fsn3.70506

**Published:** 2025-06-27

**Authors:** Moein Askarpour, Bahia Namavar Jahromi, Mohammad Hadi Eskandari, Zahra Maghsoudi, Mandana Famouri, Alireza Bedeltavana, Najmeh Hejazi

**Affiliations:** ^1^ Department of Clinical Nutrition, School of Nutrition and Food Sciences Shiraz University of Medical Sciences Shiraz Iran; ^2^ Students' Research Committee, School of Nutrition and Food Science Shiraz University of Medical Sciences Shiraz Iran; ^3^ Infertility Research Center Shiraz University of Medical Sciences Shiraz Iran; ^4^ Department of OB‐GYN, School of Medicine Shiraz University of Medical Sciences Shiraz Iran; ^5^ Department of Food Science and Technology, School of Agriculture Shiraz University Shiraz Iran; ^6^ Iranian Social Security Organization Isfahan Province Health Administration Isfahan Iran; ^7^ Dairy Expert at Research and Development of Zarrin Ghazal Company (DAITY) Shiraz Iran

**Keywords:** functional food, polycystic ovary syndrome, probiotics, vitamin D, vitamin E

## Abstract

This study aimed to determine the effects of daily consumption of fortified yogurt on the metabolic health parameters in women with PCOS. In this randomized double‐blind controlled trial, participants received either yogurt fortified with 10^6^ CFU/g probiotics, 1000 IU vitamin D, and 50 IU vitamin E in the intervention group (*n* = 45) or plain yogurt in the control group (*n* = 45) for 8 weeks. The group that consumed fortified yogurt demonstrated a significant reduction in homeostatic model assessment for insulin resistance (HOMA‐IR) (*p* = 0.040), high‐sensitivity C‐reactive protein (Hs‐CRP) (*p* = 0.012), malondialdehyde (*p* = 0.020), and total testosterone (*p* = 0.042), along with an increase in serum levels of 25(OH) D (*p* = 0.003) and alpha‐tocopherol (*p* = 0.020) compared to the control group. Nevertheless, further research is required to confirm the efficacy of this functional food as an adjunctive therapy for addressing metabolic health issues in PCOS patients.

**Trial Registration:** The Iranian Registry of Clinical Trials (IRCT20231210060323N1)

## Graphical abstract

- Polycystic ovary syndrome (PCOS) is the leading cause of anovulation worldwide.
- The present randomized controlled clinical trial was conducted on PCOS patients to assess the efficacy of a novel fortified yogurt.
- First trial showing the synergetic effect of probiotics with vitamin D and vitamin E.
- The fortified yogurt as a non‐invasive complementary therapy improved PCOS symptoms.

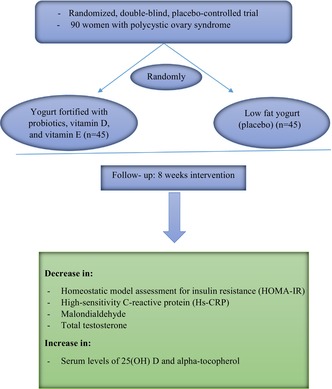

## 1 Introduction

Polycystic ovary syndrome (PCOS), a complex reproductive disorder, affects approximately 10% of women aged 18–45 years worldwide, making it the most prevalent endocrine disorder leading to anovulatory infertility among women of reproductive age (Salari et al. 2024). According to the Rotterdam criteria, PCOS is defined by irregular menstrual cycles, increased androgen levels, and/or the presence of small cysts on one or both ovaries (“Revised 2003 consensus on diagnostic criteria and long‐term health risks related to polycystic ovary syndrome,” 2004). Women with PCOS experience a range of complications, including obesity, acne, amenorrhea, excessive hair growth, and infertility. As a global health concern, PCOS can also increase the risk of endometrial cancer, cardiovascular disease, dyslipidemia, and type 2 diabetes mellitus (Ndefo et al. 2013; Norman and Teede 2018).

To alleviate PCOS symptoms, numerous therapeutic strategies and clinical guidelines have targeted insulin resistance as the underlying cause of PCOS (Rocha et al. 2019). Currently, weight reduction and the use of insulin‐sensitizing agents are recommended to enhance insulin sensitivity in PCOS patients (Li, Chi, et al. 2022). Furthermore, recent studies have highlighted dietary interventions as complementary strategies for managing insulin resistance in PCOS, aiming to improve both endocrine and metabolic health (Alesi et al. 2022; Faghfoori et al. 2017).

Recent research confirms the potential effects of micronutrients such as vitamins D and E, as well as probiotics, in improving insulin resistance (Asbaghi et al. 2023; Bagarolli et al. 2017; Sung et al. 2012). Furthermore, supplementation with either vitamin D, vitamin E, or probiotics has gained notable attention for its positive impacts on PCOS symptoms by improving gut microbiota, which plays a crucial role in the pathogenesis of PCOS (Corrie et al. 2023; Mohan et al. 2023; Singh et al. 2020). Beyond these beneficial impacts, the substantial anti‐inflammatory properties of these nutritional factors may significantly contribute to weight loss, hormonal balance, and improved metabolic profiles in women with PCOS (Tabrizi et al. 2022; Tefagh et al. 2022; Zhao et al. 2021). Therefore, combining vitamin D, vitamin E, and probiotics appears to be a promising potential treatment for PCOS.

Due to the high prevalence and significant complications of PCOS, the importance of nutritional interventions, and the beneficial effects of probiotics, vitamins D and E, a novel dairy product fortified with these nutrients was developed to address reported deficiencies and lack of multi‐ingredient nutritional interventions in PCOS patients. To the best of our knowledge, no prior research has delved into the synergistic effects of combining vitamins D and E with probiotics, nor investigated the impact of a fortified dairy product on PCOS patients. Therefore, this randomized controlled trial aimed to assess the efficacy of yogurt enriched with probiotics, vitamins D and E on weight loss, endocrine hormones, and metabolic profiles of women with PCOS.

## 2 Materials and Methods

### 2.1 Study Design and Population

This study was a parallel, randomized, double‐blind, placebo‐controlled clinical trial conducted in accordance with the Declaration of Helsinki and good clinical practice guidelines. The trial took place at the infertility clinic of Hazrat Zeinab Hospital, affiliated with Shiraz University of Medical Sciences, Shiraz, Iran. The study protocol received approval from the Ethics Committee of Shiraz University of Medical Sciences (IR.SUMS.SCHEANUT.REC.1402.104) and was registered with the Iranian Registry of Clinical Trials (IRCT20231210060323N1).

The study included women aged 18–45 with a PCOS diagnosis based on the Rotterdam criteria (18), all of whom were referred to the infertility clinic. In this study, patient eligibility was assessed by the gynecologist (BNJ) at the infertility clinics. Participant screening took place from January to March 2024 to minimize cutaneous vitamin D synthesis. The study protocol was thoroughly explained to all participants, and they provided written informed consent prior to enrollment. Participants were excluded from the study if they met the following criteria: Participants were excluded from the study if they met the following criteria: (1) non‐adherence to the study protocol; (2) engagement in a specific physical activity program or diet within three months prior to or during the study period; (3) consumption of medications or any nutritional supplements affecting health parameters (i.e., agents potentially influencing metabolic, hormonal, inflammatory, or oxidative markers, such as metformin, corticosteroids, hormonal therapies, or any type of dietary supplements) within three months before or during the study; (4) pregnancy or lactation; (5) smoking or regular alcohol consumption; (6) allergies to probiotics or dairy products; (7) daily use of probiotic products; and (8) severe medical conditions other than PCOS (including chronic or acute diseases such as diabetes, cardiovascular, hepatic, renal, autoimmune, oncologic, or major psychiatric disorders, based on clinical history and physician assessment).

### 2.2 Sample Size

The sample size was calculated utilizing G‐Power software (version 3.1.9.4), informed by the observed reduction in HOMA‐IR levels (the primary outcome) from a prior study (Ahmadi et al. 2017). To determine the sample size, an effect size (d) of 0.66, a significance level (α) of 0.05, a statistical power (1−β error probability) of 0.80, and an equal allocation ratio between the two groups (N2/N1 = 1) were considered. Based on the initial calculations, it was determined that 38 participants per group were necessary. To accommodate a potential 20% dropout rate, a total of 45 subjects were included in each arm of the study, resulting in a combined total of 90 participants.

### 2.3 Randomization and Blinding

An independent statistician, uninvolved in the data collection, devised the randomization scheme. Using Balanced Blocked Randomization (BBR) with a fixed block size of 4 and an allocation ratio of 1:1, the randomization was generated. Allocation was completed prior to the study's commencement by assigning treatment orders to participants. The code assignments were kept confidential until the completion of the statistical analyses. All group assignments were concealed from interdisciplinary researchers, including care providers, dietitians, and technicians, as well as the participants.

The two yogurt variants (LY and FY) were supplied in identical packaging by Zarin Ghazal Dairy Industries Co. (DAITY), Shiraz, Iran. Furthermore, the yogurts were indistinguishable in terms of color, appearance, smell, and taste. To ensure blinding of participants and researchers with respect to interventions and group allocation, the products were labeled as A and B.

### 2.4 Interventions

To identify suitable participants for inclusion in the study and to mitigate confounding effects, both groups underwent a 2‐week run‐in period. During this time, they were advised to maintain their usual diets and physical activities. Following the run‐in period, the participants were randomly assigned to one of the two study groups: (1) the intervention group (*n* = 45), which received 120 g of fortified yogurt (containing ≥ 10^6^ cfu/g Bb‐12 and LA‐5, 1000 IU vitamin D, and 50 IU vitamin E), and (2) the control group (*n* = 45), which received 120 g of low‐fat conventional yogurt. The participants were directed to include the yogurt in their daily meals, either at lunch or dinner, throughout the study. Yogurts were delivered weekly in insulated containers with frozen gel packs to maintain cold chain integrity, and participants were advised to store the yogurt packs in a refrigerator at temperatures below 4°C. Additionally, participants were encouraged to continue their regular physical activities, dietary habits, and lifestyle routines during the study period.

The dosage was carefully selected to safely increase and sustain circulating serum levels of 25(OH)D and α‐tocopherol over an 8‐week period (Hager et al. 2019; Nikooyeh et al. 2011), aligning closely with the recommended daily allowance (RDA) rather than the higher doses often prescribed for PCOS patients (Dastorani et al. 2018; Fatemi et al. 2017). Additionally, yogurt was chosen for fortification due to its broad acceptance, nutritional value, cost‐effectiveness, and appeal, especially among adults (Bayarri et al. 2010). The selected probiotic strains, *Bifidobacterium animalis* Bb‐12 and *Lactobacillus acidophilus* LA‐5, were chosen based on their proven stability and efficacy in yogurt‐based products, and their beneficial effects on metabolic health in clinical studies (Rezazadeh et al. 2021; Savard et al. 2011).

The low‐fat yogurt (LY) was made with starter cultures of S. thermophiles and *L. bulgaricus* , while the fortified yogurt (FY) contained these same starter cultures plus an enrichment of at least 10^6^ cfu/g of *Bifidobacterium animalis* Bb‐12 and *Lactobacillus acidophilus* LA‐5 (Chr. Hansen, Hoersholm, Denmark). During the final stages of yogurt production, once a pH of 4.7 was reached, vitamin D3 powder (product code: 5012015; DSM Nutritional Products Ltd., Basel, Switzerland) and vitamin E in the form of alpha‐tocopheryl acetate (product code: 5012740; DSM Nutritional Products Ltd., Basel, Switzerland) were incorporated and evenly distributed throughout the product.

The concentration and stability of vitamins E and D in both fortified and control yogurt samples were evaluated using high‐performance liquid chromatography (HPLC) on days 1 and 7 of storage. Additional details are available in a separate publication (Askarpour et al. 2025). The final concentration of vitamins incorporated into the fortified yogurt was adjusted to compensate for any losses over the 1‐week storage period, ensuring the targeted levels of 1000 IU of vitamin D and 50 IU of vitamin E per 120 g serving. Moreover, microbiological analysis demonstrated that the average viable total count of probiotics remained above the recommended minimum threshold of 10^6^ cfu/g for both Bb‐12 and LA‐5 over a two‐week period.

### 2.5 Compliance Assessment

A weekly self‐reported dairy intake checklist was applied to monitor participants' adherence. To document yogurt consumption, the participants filled in empty boxes on the checklist after each meal. Additionally, daily reminders via short messages were sent to encourage yogurt intake, and participants returned the empty yogurt packs during their weekly visits for adherence assessment. Side effects encountered during the intervention were discussed in weekly meetings, and participants who reported significant adverse effects were excluded from the study. A consumption rate of at least 80% of the provided product was deemed acceptable.

### 2.6 Primary and Secondary Outcomes

In this study, the primary outcome was HOMA‐IR, while other measured variables were considered as secondary.

### 2.7 Demographic Assessments

At the outset of the study, a comprehensive questionnaire was administered to collect demographic information. This included details on age, education, marital status, occupation, health conditions, family history of PCOS, daily sun exposure, and the use of any medications or dietary supplements. Specifically, daily sun exposure was recorded as the self‐reported average number of minutes per day that participants were exposed to direct sunlight between 10 a.m. and 4 p.m., with uncovered hands and face.

### 2.8 Anthropometric Assessments

Participants' height was measured with precision using a stadiometer (Seca, Germany), accurate to the nearest 0.1 cm, as they stood barefoot with their shoulders, buttocks, and heels touching the wall, and their head positioned according to the Frankfurt plane. Weight, lean body mass, body fat percentage, and body mass index (BMI) were assessed using a bioelectrical impedance analysis (BIA) device (Tanita BC‐418, Japan). Participants wore minimal clothing, maintained normal hydration, and abstained from vigorous exercise for at least 12 h before undergoing body composition assessments using BIA. Waist circumference (WC) was measured to the nearest 0.1 cm using an inelastic tape. The measurement was taken at the narrowest part between the last rib and the iliac crest while participants stood upright. Hip circumference (HC) was measured at the point of maximum buttock protrusion, with participants standing erect and feet together. The waist‐to‐hip ratio (WHR) is determined by dividing the WC by the HC.

### 2.9 Blood Pressure Assessment

Prior to taking blood pressure measurements, patients were advised to sit and rest for 5 min. The patient's right arm was positioned horizontally, extended, and aligned with the heart. A health care professional then measured blood pressure twice on the patient's right arm using a sphygmomanometer (Riester Precisa‐N, Germany), and the average of the two readings was recorded.

### 2.10 Dietary Intake Assessment

An expert dietitian gathered data on participants' dietary intake at the start and end of the intervention through a 3‐day food recall that included two regular weekdays and a weekend day. The food records were then converted to grams using standard household scales for Iranian cuisine to assess energy and nutrient content. The data was analyzed using Nutritionist 4 software (First Databank Inc., San Bruno, CA, USA), specifically designed for Iranian foods.

### 2.11 Physical Activity Assessment

The International Physical Activity Questionnaire Short Form (IPAQ‐SF), which includes seven questions, was employed to evaluate participants' physical activity levels both before and after the study (Lee et al. 2011). These questions address the intensity of physical activity (light, moderate, vigorous) and its duration in minutes over a week. Physical activity was quantified by multiplying the intensity (MET) by the duration (minutes) of the activity, yielding MET minutes per week.

### 2.12 Hirsutism and Acne Assessments

At both the start and end of the study, a gynecologist assessed hirsutism and acne severity. The Investigator Global Assessment (IGA) questionnaire and the modified Ferriman–Gallwey questionnaire were used to measure acne and hirsutism scores, respectively, before and at the end of the intervention (Kolodziejczyk et al. 2000; Lumezi et al. 2018). For hirsutism, each area was rated from 0 (no excessive terminal hair growth) to 4 (excessive terminal hair growth), and the scores from all nine areas were summed up. Moreover, acne severity was measured by the global acne grading system, which grades severity as 0 (no acne), 1 (mild acne on the face only), 2 (moderate acne), and 3 (severe acne on the face, back, or chest).

### 2.13 Biochemical Assessments

Following a 10‐h overnight fast, each participant provided a 6 mL venous blood sample. The serum was extracted through centrifugation at 3000 rpm for 5 min and subsequently stored at −75°C. Serum insulin concentrations were determined using commercial Enzyme‐Linked Immunosorbent Assay (ELISA) kits (Monobind, USA). To assess fasting blood glucose (FBS), high‐density lipoprotein (HDL), low‐density lipoprotein (LDL), triglycerides (TG), and total cholesterol (TC), we utilized commercial kits (Pars Azmun, Iran) along with the automated colorimetric methods (auto‐analyzer BT‐1500, Italy). High‐sensitive C‐reactive protein (Hs‐CRP) levels in serum were measured using a commercial CRP enzyme immunoassay kit (Diagnostic Biochem Canada (DBC), Canada). Serum total antioxidant capacity (TAC) and malondialdehyde (MDA) levels were assessed using the ELISA method with commercial kits (Zellbio, Germany). ELISA kits (Pars Azmun, Iran) were employed to assess serum levels of follicular‐stimulating hormone (FSH) and luteinizing hormone (LH). Serum levels of sex hormone binding globulin (SHBG), dehydroepiandrosterone sulfate (DHEAS), and total testosterone were determined by ELISA kits (Monobind, USA). The homoeostasis model of assessment‐insulin resistance (HOMA‐IR), homeostasis model of assessment β‐cell function (HOMA‐β), quantitative insulin sensitivity check index (QUICKI), free androgen Index (FAI), and atherogenic index of plasma (AIP) were calculated based on their suggested formulas (Al Kindi et al. 2012; Dobiásová and Frohlich 2001; Hrebícek et al. 2002). To determine the total serum 25‐hydroxyvitamin D (25(OH)D) concentration, we employed ELISA kits (LDS, USA). Additionally, alpha‐tocopherol concentrations in plasma samples were measured using the alpha‐tocopherol ELISA Kit (US Biological, USA).

### 2.14 Statistical Analyses

The Kolmogorov test was used to evaluate the normal distribution of data. Quantitative variables were reported as mean (standard error, SE), while qualitative variables were presented as numbers (%). Baseline characteristics between the intervention and control groups were compared using an independent *t*‐test for quantitative variables and the chi‐squared test for qualitative variables. Within‐group differences were examined using the paired *t*‐test for normally distributed data or the Wilcoxon rank‐sum test for non‐normally distributed data. Between‐group differences were evaluated using the independent *t*‐test for normally distributed data or the Mann–Whitney *U* test for skewed data. An analysis of covariance test was used to measure the effect of the 8‐week intervention by adjusting the baseline values as covariates. All statistical analyses were conducted using IBM SPSS Statistics version 25. A *p*‐value less than 0.05 was considered statistically significant.

## 3 Results

**FIGURE 1:**
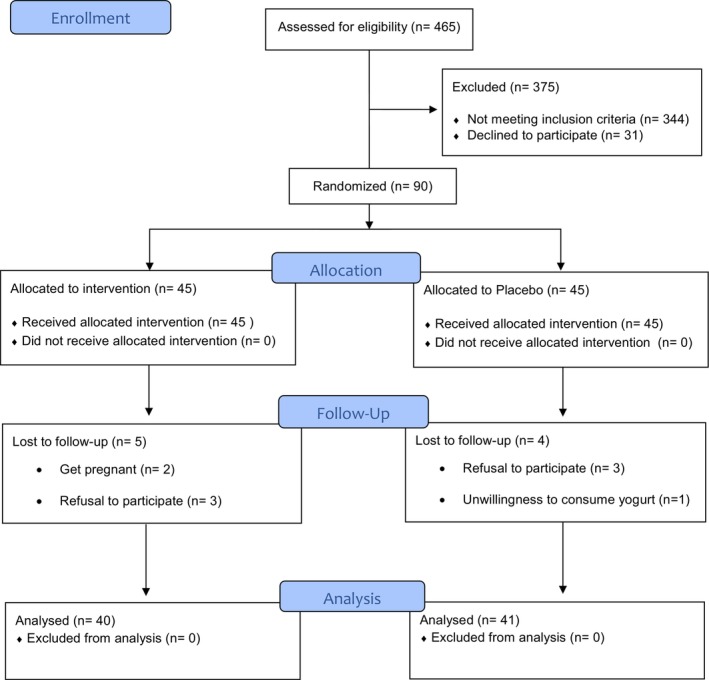
Flowchart of study design and participants (CONSORT flow diagram).

The composition of the two types of yogurt (FY and LY) is detailed in Table S1. From the initial screening of 465 patients assessed for eligibility, 90 women diagnosed with PCOS were selected for the study. All 90 participants completed a 2‐week run‐in period. Throughout the study, 9 participants were excluded, comprising 4 from the intervention group and 5 from the control group, due to refusal to continue, pregnancy, or reluctance to consume dairy products. Ultimately, 81 participants completed the 8‐week intervention period (Figure 1). No adverse effects were reported by the participants from the consumption of the yogurt.

There were no significant differences between the study groups (*p* > 0.05) in terms of baseline characteristics including demographic information, anthropometric measurements, endocrine hormones, acne and hirsutism scores, lipid profile, glycemic indices, inflammatory and antioxidant markers, blood pressure, daily sun exposure, physical activity, or serum levels of vitamins D and E (Table 1). The alterations in dietary intake, daily sun exposure, and physical activity levels did not exhibit significant differences between the groups throughout the study (*p* > 0.05) (Table S2).

**TABLE 1:** Baseline characteristics of the participants.

Variables	Control group, *n* = 45	Intervention group, *n* = 45	*p*
Age (year)	33.42 (0.82)	33.46 (0.81)	0.971
Family history of PCOS, *n* (%)*	5 (11.1)	7 (15.6)	0.535
Married, *n* (%)*	43 (95.6)	43 (95.6)	0.992
Diploma and upper, *n* (%)*	37 (88.2)	36 (80)	0.783
Employed, *n* (%)*	10 (22.2)	12 (26.7)	0.621
Weight (kg)	74.46 (1.95)	74.27 (1.87)	0.940
Body mass index (kg/m^2^)	28.66 (0.72)	28.63 (0.71)	0.974
Waist circumference (cm)	82.38 (1.87)	85.91 (2.14)	0.211
Hip circumference (cm)	92.06 (2.02)	92.46 (2.05)	0.892
WHR	0.91 (0.002)	0.92 (0.007)	0.110
Lean body mass (kg)	38.95 (1.05)	38.85 (1.58)	0.631
Fat mass (%)	34.08 (0.86)	36.13 (1.27)	0.181
Total testosterone (ng/mL)	0.62 (0.06)	0.79 (0.08)	0.122
LH (IU/L)	8.45 (1.09)	8.93 (1.05)	0.751
FSH (IU/L)	9.15 (1.26)	13.22 (2.46)	0.141
SHBG (nmol/L)	45.30 (2.55)	48.93 (2.67)	0.330
FAI	0.05 (0.005)	0.06 (0.007)	0.375
DHEAS (μg/mL)	1.06 (0.08)	1.21 (0.08)	0.190
Acne score	1.4 (0.16)	1.6 (0.17)	0.245
Hirsutism score	14.95 (0.83)	15.06 (0.95)	0.935
Hs‐CRP (mg/L)	3.28 (0.24)	3.55 (0.25)	0.454
MDA (μmol/L)	3.26 (0.14)	3.09 (0.14)	0.410
TAC (mmol/L)	1.50 (0.04)	1.47 (0.04)	0.595
FBS (mg/dL)	91.67 (1.79)	88.82 (2.10)	0.302
Insulin (μIU/mL)	8.26 (0.79)	7.67 (0.56)	0.542
HOMA‐IR	1.91 (0.20)	1.70 (0.14)	0.385
HOMA‐β	8.08 (0.46)	8.77 (1.36)	0.696
QUICKI	0.30 (0.001)	0.30 (0.001)	0.615
TG (mg/dL)	104.05 (5.57)	108.19 (6.34)	0.622
TC (mg/dL)	180.80 (5.03)	165.34 (6.63)	0.101
LDL (mg/dL)	106.47 (3.27)	105.80 (2.79)	0.875
HDL (mg/dL)	35.65 (0.96)	34.92 (1.26)	0.654
AIP	1.02 (0.06)	1.08 (0.07)	0.585
Systolic blood pressure (mmHg)	118.02 (2.18)	120.24 (1.80)	0.436
Diastolic blood pressure (mmHg)	76.20 (1.93)	80.04 (1.72)	0.145
Serum 25 (OH) D (ng/mL)	30.54 (2.48)	27.50 (2.28)	0.394
Serum alpha‐tocopherol (mg/L)	8.57 (0.33)	8.82 (0.35)	0.633
Sun exposure (minute/day)	26 (2.38)	24 (2.08)	0.425
Physical activity (MET min/week)	555.28 (32.79)	557.15 (30.21)	0.964

*Note:* *p*‐value from Independent samples *t*‐test (parametric variables with normal distribution); *Chi‐square test (non‐parametric variables).

Abbreviations: AIP, atherogenic index of plasma; DHEAS, dehydroepiandrosterone sulfate; FAI, free androgen index; FBS, fasting blood sugar; FSH, follicle‐stimulating hormone; HDL, high density lipoprotein; HOMA‐β, homeostasis model assessment of β‐cell function; HOMA‐IR, homeostatic model assessment for insulin resistance; Hs‐CRP, high‐sensitivity C‐reactive protein; LDL, low density lipoprotein; LH, luteinizing hormone; MDA, malondialdehyde; QUICKI, quantitative insulin sensitivity check index; SHBG, sex hormone binding globulin; TAC, total antioxidant capacity; TC, total cholesterol; TG, triglyceride; WHR, waist‐to‐hip ratio.

* Values are expressed as number (percentage of participants in each group) and other variables are presented as mean (SE).

The primary findings indicated that, within the intervention group, the consumption of fortified yogurt led to a significant reduction in total testosterone (*p* = 0.04), LH (*p* = 0.01), acne score (*p* = 0.03), hirsutism score (*p* = 0.01), Hs‐CRP (*p* = 0.03), MDA (*p* < 0.001), and TC (*p* = 0.002). Concurrently, there was an increase in SHBG (*p* = 0.03), TAC (*p* = 0.009), and serum levels of 25(OH) D (*p* = 0.001) and alpha‐tocopherol (*p* = 0.005) in the intervention group (Table 2).

**TABLE 2:** The effects of the intervention on the measured variables.

Variables	Control group, (*n* = 41)		Intervention group, (*n* = 40)		Intervention effect
	Change	Intragroup *p* *	Change	Intragroup *p* *	*p* ^¥^
Weight (kg)	0.27 (0.32)	0.41	−0.26 (0.34)	0.44	0.264
Body mass index (kg/m^2^)	0.11 (0.13)	0.38	−0.09 (0.13)	0.47	0.263
Waist circumference (cm)	−0.37 (0.28)	0.89	0.19 (0.49)	0.69	0.687
Hip circumference (cm)	−0.32 (0.33)	0.33	0.19 (0.56)	0.72	0.428
WHR	0.003 (0.004)	0.35	0.002 (0.004)	0.51	0.260
Lean body mass (kg)	−1.58 (0.55)	0.05	−1.36 (1.72)	0.43	0.901
Fat mass (%)	0.41 (0.71)	0.56	−0.50 (0.67)	0.45	0.355
Total testosterone (ng/mL)	0.06 (0.45)	0.38	−0.15 (0.55)	**0.04**	**0.042**
LH (IU/L)	−1.52 (1.66)	0.36	−2.61 (1.05)	**0.01**	0.585
FSH (IU/L)	−1.95 (1.40)	0.17	−2.61 (1.05)	0.07	0.474
SHBG (nmol/L)	2.96 (3.79)	0.43	7.32 (3.36)	**0.03**	0.393
FAI	0.005 (0.01)	0.60	−0.01 (0.008)	0.14	0.191
DHEAS (μg/mL)	−0.05 (0.10)	0.58	−0.07 (0.12)	0.55	0.912
Acne score	−0.25 (0.09)	0.10	−0.43 (0.11)	**0.01**	0.204
Hirsutism score	−0.77 (0.46)	0.10	−0.87 (0.40)	**0.03**	0.867
Hs‐CRP (mg/L)	−0.02 (0.31)	0.94	−1.12 (0.29)	**0.004**	**0.012**
MDA (μmol/L)	−0.35 (0.18)	0.07	−0.94 (0.16)	**< 0.001**	**0.020**
TAC (mmol/L)	0.01 (0.05)	0.78	0.19 (0.06)	**0.009**	0.064
FBS (mg/dL)	−2.85 (2.03)	0.16	−2.95 (1.77)	0.10	0.971
Insulin (μIU/mL)	1.24 (1.12)	0.27	−0.63 (0.68)	0.35	0.156
HOMA‐IR	0.18 (0.27)	0.51	−0.45 (0.14)	0.17	**0.040**
HOMA‐β	1.01 (0.96)	0.24	5.63 (3.61)	0.12	0.491
QUICKI	−0.003 (0.002)	0.19	0.002 (0.002)	0.24	0.073
TG (mg/dL)	−1.47 (6.15)	0.81	−6.29 (5.06)	0.22	0.545
TC (mg/dL)	−17.92 (6.67)	0.11	−22.80 (7.06)	**0.002**	0.611
LDL (mg/dL)	−5.05 (4.63)	0.28	−5.80 (5.82)	0.32	0.922
HDL (mg/dL)	0.42 (1.46)	0.77	2.29 (1.65)	0.17	0.401
AIP	−0.16 (0.09)	0.10	−0.14 (0.07)	0.05	0.871
Systolic blood pressure (mmHg)	5.40 (2.98)	0.18	3.41 (2.45)	0.17	0.603
Diastolic blood pressure (mmHg)	5.20 (2.85)	0.14	2.58 (2.30)	0.26	0.085
Serum 25 (OH) D (ng/mL)	−1.04 (1.23)	0.40	4.55 (1.30)	**0.001**	**0.003**
Serum alpha‐tocopherol (mg/L)	−0.33 (2.49)	0.41	0.78 (1.68)	**0.005**	**0.020**

*Note:* Data expressed as mean (SE).

Abbreviations: AIP, atherogenic index of plasma; DHEAS, dehydroepiandrosterone sulfate; FAI, free androgen index; FBS, fasting blood sugar; FSH, follicle‐stimulating hormone; HDL, high density lipoprotein; HOMA‐β, homeostasis model assessment of β‐cell function; HOMA‐IR, homeostatic model assessment for insulin resistance; Hs‐CRP, high‐sensitivity C‐reactive protein; LDL, low density lipoprotein; LH, luteinizing hormone; MDA, malondialdehyde; QUICKI, quantitative insulin sensitivity check index; SHBG, sex hormone binding globulin; TAC, total antioxidant capacity; TC, total cholesterol; TG, triglyceride; WHR, waist‐to‐hip ratio.

* *p* values denote the significance of within‐group changes (Paired samples *t*‐test). *p*‐value less than 0.05 is considered statistically significant.

^¥^ *p* values denote the significance of between‐group changes (Analysis of covariance, adjusted for baseline values). *p*‐value less than 0.05 is considered statistically significant.

However, in the control group, there were no statistically significant changes detected in the measured variables over time. The results of the between‐group comparison showed that, compared to the control group, the intervention group experienced a significant reduction in total testosterone (*p* = 0.042), Hs‐CRP (*p* = 0.012), MDA (*p* = 0.020), and HOMA‐IR (*p* = 0.040). Additionally, there was a notable increase in serum levels of 25(OH) D (*p* = 0.003) and alpha‐tocopherol (*p* = 0.020) (Table 2).

## 4 Discussion

To our knowledge, this is the first comprehensive study exploring the potential benefits of daily consumption of a novel fortified yogurt enriched with probiotics, vitamin D, and vitamin E on various health parameters such as weight indices, endocrine hormones, inflammatory and antioxidant markers, insulin resistance, lipid profiles, and blood pressure in PCOS patients. This randomized controlled trial revealed that daily consumption of yogurt fortified with probiotics, vitamin D, and vitamin E over an eight‐week period significantly reduced HOMA‐IR, Hs‐CRP, MDA, and total testosterone levels in women with PCOS. However, while the intervention group demonstrated significant within‐group improvements in LH, SHBG, TAC, TC, acne, and hirsutism scores, these changes did not reach statistical significance compared to the control group over the study duration. Nonetheless, these non‐statistically significant changes may still hold clinical importance. The important finding of our study is that including a functional food with safe doses of probiotics, as well as vitamins D and E, in the participants' diet (rather than the high doses typically prescribed for each nutrient separately) can improve the health parameters in PCOS patients.

Despite PCOS widespread occurrence and significant impact on reproductive, metabolic, and psychological health, the importance of preventing PCOS is often underestimated. This is partly due to the fact that many women remain undiagnosed and unaware of their condition until they seek treatment for infertility (Dennett and Simon 2015). Lifestyle changes, especially dietary modifications, are considered as the foremost preventive approach for preventing PCOS and improving its complications. Nonetheless, no specific dietary composition has been definitively identified as the most effective to date, largely due to clinical heterogeneity and methodological issues (Li, Ruan, and Mueck 2022). Over recent decades, numerous studies have explored various dietary approaches to alleviate PCOS symptoms. Among these, supplementation with probiotics, vitamin D, and E has become particularly widespread due to their beneficial effects.

Ostadmohammadi et al. conducted a clinical study examining co‐supplementation effects on PCOS patients, revealing that a 12‐week co‐administration of vitamin D (50,000 IU/per two weeks) and probiotics (8 × 10^9^ CFU/day) had positive effects on serum total testosterone, Hs‐CRP, TAC, and MDA levels (Ostadmohammadi et al. 2019). Additionally, they observed no positive effects on weight loss measurements, which align with our findings. Although there is no study exploring the synergistic effects of probiotics with vitamins D and E, the effects of each have been individually assessed in previous studies.

In a meta‐analysis study, Tabrizi et al. found that probiotic supplementation improved weight indices, insulin resistance, lipid profiles, inflammatory markers, and SHBG levels. However, it did not affect DHEAS or TC, LDL, and HDL levels in PCOS patients (Tabrizi et al. 2022). This meta‐analysis indicated that supplementing with specific probiotics such as *Lactobacilli* and *Bifidobacteria*, which likely counteract their reduction in the gut microbiome of PCOS patients (LA‐5 and BB‐12 were used in our study), led to a more positive impact on PCOS symptoms (Calcaterra et al. 2023).

Concerning the effect of vitamin D on metabolic profiles, Miao et al. found that supplementation with vitamin D failed to show a beneficial impact on BMI, DHEAS, TG levels, or HDL. However, it was effective in reducing hyperandrogenism and insulin resistance, as well as improving TC in patients with PCOS (Miao et al. 2020). The beneficial effects have been reported with high doses of vitamin D supplementation over extended periods (Cochrane et al. 2023). However, some studies suggest that these benefits may be attributed to serum levels of 25(OH)D rather than the duration or dosage of supplementation (He et al. 2015). It is noteworthy that, in our study, we observed no differences between the two groups concerning baseline serum levels of vitamins D and E, therefore, we cannot conclusively dismiss the hypothesis of the efficacy of supplementation dosage irrespective of serum values.

In addition to vitamin D, the impact of vitamin E on metabolic profiles and infertility in PCOS patients has been explored in prior studies. Tefagh et al. demonstrated that vitamin E supplementation improves lipid profiles, reduces insulin and HOMA‐IR levels, and positively impacts metabolic and hormonal parameters in women with PCOS (Tefagh et al. 2022). However, a retrospective cohort study revealed that short‐term vitamin E supplementation in infertile women with PCOS undergoing ovulation induction did not positively affect pregnancy rates (Chen et al. 2020). These conflicting results from supplementation might be due to variations in study duration, dosage, sample size, and carriers used for administration (food or capsule).

The precise mechanisms behind the observed improvements in the outcomes of interest from the synergistic effect of vitamins D and E combined with probiotics in the fortified yogurt remain unclear, although some molecular mechanisms have been proposed for the effects of vitamins D and E as well as probiotics. The beneficial effects of vitamins D and E (fat‐soluble antioxidants) can be attributed to several mechanisms. These include suppressing the generation of reactive oxygen species (ROS) in the pancreas, maintaining the integrity of pancreatic cells, stimulating insulin receptor expression to enhance insulin sensitivity, inhibiting NF‐ĸB to reduce free radicals and pro‐inflammatory cytokines, and increasing aromatase activity to improve hormonal balance (Liao et al. 2022; Mohan et al. 2023). Moreover, probiotics may improve health parameters in PCOS patients by enhancing intestinal barrier protection and producing short‐chain fatty acid metabolites, which help balance androgen levels, reduce insulin resistance, and decrease inflammation (Angoorani et al. 2023).

The strengths of this study include pioneering the combined effects of vitamin D, vitamin E, and probiotics through a novel functional food; using fortified yogurt as a safe adjunctive therapy, without adverse effects in PCOS participants, to assess its impact on metabolic health parameters; achieving high adherence to the study protocol, confirmed by diary checklists and increased serum levels of vitamins D and E in the intervention group; and maintaining consistent dietary intake and physical activity in both groups, effectively minimizing the potential Hawthorne effect.

The primary limitation of this study was its relatively short duration. Responses to the questionnaires relied on participants' memory, which may introduce recall bias. Moreover, the multiple components added to the yogurt make it challenging to ascertain which specific components were most effective in alleviating PCOS symptoms. Moreover, budget constraints prevented the measurement of changes in gut microbiota. Participants' physical activity was measured using the IPAC questionnaire instead of an accelerometer, which would have offered more precision. Consequently, these limitations imply that the study's findings should not be generalized to the entire population of PCOS patients.

## 5 Conclusion

This study showed that combining probiotics with vitamins D and E in a novel fortified yogurt effectively improved insulin resistance, testosterone level, inflammatory and antioxidant markers in PCOS patients. The findings suggest that proper dietary interventions, as crucial preventive strategies, could serve as effective adjunctive therapy for addressing metabolic health issues in PCOS patients. However, due to the study's limitations, further research, particularly clinical studies with longer intervention periods in diverse populations, is required to confirm the beneficial effects of the fortified yogurt in women with PCOS.

## Author Contributions

**Moein Askarpour:** formal analysis; writing original draft; conceptualization. **Bahia Namavar Jahromi:** supervision. **Mohammad Hadi Eskandari:** conceptualization. **Zahra Maghsoudi:** writing. **Mandana Famouri:** conceptualization. **Alireza Bedeltavana:** conceptualization. **Najmeh Hejazi:** supervision.

## Supporting information


**Tables S1‐S2**.

## Data Availability

Data described in the manuscript will be made available upon reasonable request by the corresponding author.
