# Supplementary material for: The Effects of a Novel Fortified Dairy Product on Weight Loss, Metabolic Profiles, and Endocrine Hormones in Women With Polycystic Ovary Syndrome: A Double‐Blind Randomized Controlled Trial
Source: Food Sci Nutr. 2025 Jun 27;13(7):e70506. doi: 10.1002/fsn3.70506 (PMC12203567; doi:10.1002/fsn3.70506)
Supplement: Supplementary file 1 — Tables S1‐S2. [file FSN3-13-e70506-s001.docx]

**Supplementary Materials**

| **Supplementary Table 1.** Nutritional composition of FY and LY per serving (120 g) | | |
| --- | --- | --- |
| **Components** | **Fortified Yogurt (FY)** | **Low-fat Yogurt (LY)** |
| Energy (kcal) | 65 | 65 |
| Carbohydrate (g) | 8 | 8.6 |
| Fat (g) | 1.65 | 1.6 |
| Protein (g) | 5.6 | 5.4 |
| Cholesterol (mg) | 2.2 | 2.2 |
| PH | 4.3 | 4.3 |
| Bifidobacterium lactis Bb12 (cfu/g) | ≥10^6^ | 0 |
| Lactobacillus acidophilus La5 (cfu/g) | ≥10^6^ | 0 |
| Vitamin D (IU) | 1000 | 0 |
| Vitamin E (IU) | 50 | 0 |

| **Supplementary Table 2.** Comparison of changes in dietary intakes, daily sun exposure, and physical activity during the study | | | |
| --- | --- | --- | --- |
| **Components** | **Control Group, (n=41)** | **Intervention Group, (n=40)** | **P value^*^** |
| Energy (kcal/day) | -77.40 (43.82) | -71.39 (47.91) | 0.920 |
| Carbohydrate (g/day) | 0.19 (7.75) | -11.83 (7.06) | 0.261 |
| Protein (g/day) | -7.01 (4.50) | -1.51 (4.25) | 0.385 |
| Fat (g/day) | -6.01 (3.41) | -2.59 (3.22) | 0.474 |
| Fiber (g/day) | 1.66 (2.01) | 0.64 (2.35) | 0.743 |
| Chol (mg/day) | 62.55 (43.01) | 45.34 (44.57) | 0.786 |
| SFA (g/day) | -0.70 (1.54) | -0.04 (1.36) | 0.745 |
| MUFA (g/day) | -2.38 (1.05) | -2.09 (0.95) | 0.832 |
| PUFA (g/day) | -3.34 (1.73) | -1.50 (2.02) | 0.498 |
| Mg (mg/day) | 9.28 (18.50) | -3.48 (18.51) | 0.622 |
| Zinc (mg/day) | 0.03 (0.75) | -0.01 (0.83) | 0.960 |
| Calcium (mg/day) | -3.94 (9.51) | -3.5 (10.67) | 0.981 |
| Vitamin B1 (mg/day) | 0.02 (0.09) | -0.03 (0.08) | 0.663 |
| Vitamin B3 (mg/day) | -0.35 (1.31) | 0.99 (1.63) | 0.525 |
| Vitamin B6 (mg/day) | 0.17 (0.19) | 0.24 (0.17) | 0.810 |
| Vitamin B9 (μg/day) | 107.91 (68.82) | 60.62 (69.37) | 0.635 |
| Vitamin B12 (μg/day) | 9.25 (7.01) | 9.01 (6.91) | 0.986 |
| Vitamin D (mg/day) | 0.04 (0.29) | 0.35 (0.26) | 0.444 |
| Vitamin E (mg/day) | -3.69 (2.49) | -0.28 (2.83) | 0.375 |
| Sun exposure (minute/day) | 3 (0.11) | 4 (0.09) | 0.541 |
| Physical activity (MET min/week) | 82.53 (195.26) | 27.30 (173.91) | 0.421 |
| Data reported as mean change (post-pre) and (SE change).  ^*^ Between-group comparison (independent samples t-test). | | | |
